# Impact of an Intervention with Wii Video Games on the Autonomy of Activities of Daily Living and Psychological–Cognitive Components in the Institutionalized Elderly

**DOI:** 10.3390/ijerph18041570

**Published:** 2021-02-07

**Authors:** Maha Jahouh, Jerónimo J. González-Bernal, Josefa González-Santos, Diego Fernández-Lázaro, Raúl Soto-Cámara, Juan Mielgo-Ayuso

**Affiliations:** 1 Department of Health Sciences, University of Burgos, 09001 Burgos, Spain; mjx0002@alu.ubu.es (M.J.); jfmielgo@ubu.es (J.M.-A.); 2 Department of Biochemistry, Molecular Biology and Physiology, Faculty of Health Sciences, Campus of Soria, University of Valladolid, 42003 Soria, Spain; diego.fernandez.lazaro@uva.es; 3 Neurobiology Research Group, Faculty of Medicine, University of Valladolid, 47005 Valladolid, Spain

**Keywords:** memory, attention, apathy, depression, anxiety, Wii, elderly, Spain

## Abstract

As people age, the risk of disease increases and deterioration becomes more noticeable. These changes can increase the risk of cognitive impairment, with negative consequences for the quality of life and the ability to perform activities of daily living (ADLs) in older people, which translate into greater dependence and loss of wellness. This study aimed to determine the impact and effectiveness of the use of the Wii^®^ game console (Nintendo Company Limited, Kyoto, Japan) on improving performance of basic and instrumental ADLs, as well as its relationship with cognitive impairment levels and mood in institutionalized older people. A longitudinal study was designed, whose study population consisted of people over 75 years of age who lived in a nursing-home or attended a day care center (*n* = 80; 45 women). Cognitive status was assessed using Lobo’s Mini-Cognitive Examination (MCE) and Global Deterioration Scale (FAST-GDS), while the psychological assessment used the Dementia Apathy Interview and Rating (DAIR), Yesavage scale for Geriatric Depression (EGD-15), and Goldberg Anxiety and Depression Scale (EADG). Differences from T1 to T2 in the control group (control; *n* = 40; 23 women; 83.25 ± 8.78 years; 76.35 ± 13.54 kg) and in the experimental group (Wii; *n* = 40; 22 women; 85.05 ± 8.63 years; 74.60 ± 13.01 kg) were evaluated using a paired Student’s *t*-test or Wilcoxon’s signed rank test, and a two-way repeated measures analysis of variance (ANOVA) test. Differences in Δ (%) and other tests at T1 and T2 were compared using the independent *t*-test or Mann–Whitney U test, with the treatment category as a fixed factor. The results showed that the Wii^®^ video console had a positive influence for older people, increasing cognitive status and levels of ADLs, and psychological status. In addition, a positive correlation between performance of ADLs and cognitive status was observed, as well as a negative correlation with the psychological status. Through a rehabilitation program with a Wii^®^ game console in the elderly, depression, anxiety and apathy levels were reduced, accompanied by an increase in memory and attention, as well as in performance of basic and instrumental ADLs.

## 1. Introduction

The world’s population is aging rapidly. As people age, the risk of disease and dependent status increases. These changes can increase the risk of cognitive impairment, with negative consequences for quality of life and the ability to perform activities of daily living (ADLs) among older people, which translates into greater dependence and loss of wellness [1,2,3].

Tasks such as feeding, grooming, and bathing are considered basic ADLs, while instrumental ADLs include complex, goal-directed, and community-focused voluntary behaviors such as ability to handle finances, problem solving, responsibility for own medications, and housekeeping [4]. Cognitive impairment and dependence in ADLs are related to an increase in mortality, even when the effects of sociodemographic or socioeconomic variables, or health conditioning factors, are controlled [5]. Furthermore, there is an interrelation between cognitive impairment and alteration of ADL performance [6,7].

Currently, new technologies are undergoing significant development. Their implementation in everyday life has become more common. A clear example of such new technologies is the use of electronic games, which are an effective resource for the improvement of cognitive skills such as attention and memory, concentration, intelligence, and creativity and, of course, problem solving [8,9].

The new generation of video games, also called exergames, require a dynamic interaction of the player through movements in order to activate the characteristics of the game. This has aroused interest in their impact in various environments, and their physical and cognitive benefits [10,11,12]. An emerging line of research on exergames, a combination of exercise with electronic games, is the study of psychological variables, especially in their maintenance [11], since a decrease in cognitive functioning is closely related to increasing age and the appearance of depressive processes, which in turn are related to degenerative diseases, such as those related to dementias [11,13].

Some recent research has used the games of the Wii^®^ video console to determine their possible benefits in different groups in rehabilitation processes and proprioceptive neuromuscular training, as well as the impact of this type of physical activity on ADLs and quality of life [14,15]. Simsek et al. found that a rehabilitation program using the Nintendo Wii^®^ is as effective as the Bobath method in recovering the functions of daily life and quality of life in patients following an acute stroke [16]. Furthermore, Uysal et al. observed an improvement in ADL scores and balance in children with spastic hemiplegic cerebral palsy [17]. In addition, Moon et al. showed improvements in balance, gait, and ADLs in a group of Parkinson’s patients after balance training with a Wii Fit^®^ balance board [18].

However, few studies have assessed the use of the Wii Fit^®^ video console on the cognitive and psychological state, and on the performance of basic and instrumental ADLs, in older people. In this sense, Monteiro et al. showed a moderate improvement in semantic memory and executive functioning in institutionalized older people after a single session with the Wii Fit^®^ game console [19]. On the other hand, Santamaria et al. obtained improvements in dynamic balance and attention in older adults after 15 sessions with the Wii Fit^®^ game console [20]. Likewise, Chesler et al., after an intervention with Wii Fit^®^, observed a decrease in depression levels and apathy [21]. However, to the best knowledge of the authors, no studies have related the effect of the use of the Wii Fit^®^ on the performance of basic and instrumental ADLs and cognitive and psychological status in a group such as the institutionalized elderly. In this group, it is estimated that between 21% and 27% of European people have cognitive impairment [22,23], and between 32% and 54% have problems performing ADLs, which can affect their physical/motor performance [7,24].

Alteration in the performance of ADLs is a consequence of the aging process. This process interacts with others and could trigger more dependent aging as a consequence of cognitive impairment. Therefore, a precise intervention is considered necessary to improve ADL performance in the institutionalized elderly, as well as various cognitive components [25,26]. Therefore, the objective of this study was to determine the impact and effectiveness of the use of the Wii^®^ game console on improving basic and instrumental ADLs, as well as its relationship with cognitive and psychological levels, in institutionalized older people.

## 2. Materials and Methods

### 2.1. Study Design: Participants

A longitudinal study with an experimental group and control group was performed. Eighty elderly people (45 women), who were institutionalized or who attended the day care center in the “Mixed Nursing Home for the Elderly Burgos I—Cortes” (Burgos, Spain), were the study population. In order to control the influence of as many variables as possible, this study was carried out in a natural context.

Being older than 75 years, being institutionalized in the nursing-home or attending the day center on a daily basis, being able to stand up with physical support, and obtaining a score equal to or greater than 10 in the Lobo’s Mini-Cognitive Examination (MCE) were the inclusion criteria. The cut-off points for a score equal to or greater than 10 on the MCE was established since it is necessary that the person, even if they have a moderate deterioration, be able to react to and follow the guidelines of the game, or have the minimum necessary intuition.

Subjects who permanently used technical aids such as wheelchairs, those with a diagnosis of cardiovascular disease, those with hearing and/or visual limitations that prevented them from using the Nintendo Wii^®^ video console, those with severely disorganized behaviors, and those with any type of medical contraindication were excluded. The last three exclusion criteria were decided by a multidisciplinary team, including the residents’ doctor and based on the reports of each of member. Consequently, it was decided to exclude people with cardiovascular disease, since some exercises require exertion and prolonged standing and some participants, due to their conditions, could not follow the development of the sessions. On the other hand, the residential center has users with mental illnesses; some were included in the research, but others by decision of the team and medical prescription were excluded when presenting maladaptive behavior, missing sessions, or not committing to the investigation.

After verifying compliance with the established inclusion criteria, participants were selected by non-probabilistic convenience sampling. Based on the voluntary character of the study, it was designed to access the available cases from the population and select the participants from among them.

The study received a favorable report from the Institutional Review Board of the University of Burgos (Protocol code 29/2019; March 2019) and was conducted in accordance with the ethical principles of the Declaration of Helsinki.

### 2.2. Procedure: Data Collection

Prior to starting the study, meetings were held with possible participants in order to present the procedure. In the first, the project, its objective, and the intervention’s exercises, as well as its voluntary nature, were explained. They were invited to participate in the study, and signed the informed consent in the case of acceptance. Later, in successive meetings, they were instructed about the video games and virtual reality, including explanations and practical demonstrations to internalize their use.

Participants were randomized to the control group (control; *n* = 40, 23 women; 83.25 ± 8.78 years) and to the experimental group (Wii; *n* = 40, 22 women; 85.05 ± 8.63 years) by an independent investigator, using the block randomization method to ensure the same number of participants in both groups. All participants underwent an initial assessment at the time of inclusion in the study (T1), and another 8 weeks later (T2), coinciding with the completion of the intervention in the Wii group. The examiner who carried out the assessment, as well as the researcher who analyzed the data statistically, were blinded with respect to the group to which the participants belonged. In addition, clear instructions were provided to the participants to not reveal the group to which they had been assigned during the assessment visits.

The intervention consisted of 20 rehabilitation sessions, developed over eight consecutive weeks, and made up of different activities with the Nintendo Wii Fit^®^ video game console. Regarding the session distribution, all participants received three sessions in one week and two sessions in the next, rotating each week cyclically until they completed all 20 sessions. These sessions were held in the nursing home, in the assembly hall, as it had a television, a chair, and the appropriate material for the Wii^®^ game console, in addition to good lighting and ventilation, and being free from distractions. The approximate duration of each session was 40–45 min. During the sessions, the participants worked on different cognitive processes, such as memory and attention. In addition, implicitly, it was intended that participants activated their muscle tone and maintained balance during the different activities.

All sessions had four fundamental parts: (1) The games to be played in the session, as well as the necessary rules to execute them correctly, were explained to the participant by the therapist. As the intervention program progressed, the participants were expected to increase their interaction with the game, progressively reducing or eliminating verbal support from the therapist. (2) An aerobic-type game such as “Step” was used as a warm-up exercise. With this activity, the person began to interact with the interface and focused their attentional processes on the game, since, for example, without this attention the participants would not be able to coordinate the right foot, the left foot, or both feet to follow the choreography of the steps. (3) The next game was played specifically to work on attention, concentration, and memory; this game is called “Nodding”. In this game, a goalkeeper throws balls or bears from both the left and right sides. The participant was required to lean to one side or the other to avoid all possible bears and head all possible balls; in other words, the participants had to swing on the same side of the ball or on the opposite side of the bear. The more balls that were headed, and the more bears that were dodged, the more cognitive processes the participant would have activated. (4) To end the session, the participants had to choose a game that they wanted to try or play for a period of 5 min. In this last phase, the video games were expected to recover their playful component. The researchers also gathered feedback on experiences, impressions, and comments on the games from the participants in this phase.

During the intervention period, all participants continued with their conventional nursing home-provided treatments and therapies, such as physical therapy, occupational therapy, and gymnastics sessions. The control group was evaluated under the same parameters and the same time points as the Wii group, with the only difference being that they did not participate in any rehabilitation program involving virtual reality.

### 2.3. Main Outcomes: Instruments

The cognitive status, the performance of ADLs, and the psychological status of the participants were the main outcomes from the study. Their state was evaluated using the following instruments and questionnaires:

- Cognitive assessment: the cognitive status of the participants was evaluated using the MCE. This is an adapted and validated version for the Spanish population of the “Mini-Mental State Examination” [27]. In this study, the 30-point version was used, instead of the 35-point version, since it has been the most widely used internationally and allows comparisons with other investigations. The test-retest reliability is 0.89, while the inter-rater reliability is 0.82. Through 11 items, this test assesses the essential cognitive functions of the participants: orientation, registration, attention and concentration, fixation and short-term memory, language, calculation, memory, nomination, repetition, compression, reading, writing, and drawing [28]. The score obtained ranges from 1 to 30; values lower than 10 indicate severe cognitive damage, values between 11 and 20 moderate cognitive damage, values between 21 and 26 mild cognitive damage, and values higher than 27 normal cognitive status.
  The Global Deterioration Scale (GDS) was also used, which allows professionals and caregivers to measure and record the cognitive, behavioral, and functional impairment of patients [29]. This scale classifies deterioration into seven stages, where 1 corresponds to the absence of deterioration and 7 to the most severe deterioration. Stage 4 or mild deterioration is characterized by patients who require help with complex tasks such as managing finances, planning a dinner, and so forth. In stage 5, or moderate deterioration, patients need help to choose adequate clothing. In stage 6, or moderately severe deterioration, patients need help to dress and bathe, and they begin to experience urinary and fecal incontinence. Although the MCE scale is already a sufficient tool, complementing its use with the GDS is useful because this tool provides information on behavioral deterioration and dependent status, and also relates the person’s cognitive status with their score on the MCE. Each stage of the GDS is related to a score of the MCE, so if a person improves or worsens, changes on his score in MCE and GDS stage can reflect this [30].
- Functional assessment: to assess the basic ADLs, the Katz Index of Independence in Activities of Daily Living was used [31]. This index assesses six basic functions in terms of dependency or independence: bathing, dressing, toileting, transferring, continence, and feeding. Its assessment is based on the direct observation of the patient by health personnel during their stay in a center, and/or by direct questioning with the patient, caregivers, or a family member by health personnel. The ability to perform each of the tasks is valued at 0, while disability is valued at 1. Therefore, the higher the score, the greater the dependency. It is an effective indicator of active life expectancy, since the higher the score, the lower the active life expectancy. It is an indicator poorly sensitive to small changes in ADL performance [32].
  To complement the assessment of basic ADLs, the Barthel Index [33] was also used. Both scales were included in the research to determine the AVDS of a patient because, while the Katz scale measures rapid changes (acute context), the Barthel scale is more conducive to the long-term assessment [34]. This index assesses the level of independence of the patient in some basic ADLs, such as feeding, bathing, grooming, dressing, bowel control, bladder control, toilet use, transferring (bed to chair and back), and mobility on level surfaces or stairs. The evaluator assigns different scores and weights according to the patient’s ability to perform the different activities. The score obtained ranges from 1 to 100, with intervals of 5 points; values closer to 0 indicate higher levels of dependency, and values closer to 100 more independence [33,35].
  The evaluation was carried out by an occupational therapist, accompanied by the auxiliary reference staff and the residential center occupational therapist. In this case, there was no interview with any family members, since the information from two different professionals was sufficient and included the nursing assistant who assists users in ADLs, and the occupational therapist who oversees evaluation and stimulation of the AVDs of the users.
  The ability to carry out instrumental ADLs was assessed using the Lawton and Brody index [36]. This index assesses eight instrumental activities, such as the ability to use a telephone, shopping, food preparation, housekeeping, laundry, mode of transportation, responsibility for own medications, and ability to handle finances. Those people with an inability to perform one or more activities are considered dependent to different degrees. Each area is scored according to the description that best corresponds with it, assigning a maximum of 1 point and a minimum of 0 points. The maximum dependence would be reflected by a score of 0 points, while a score of 8 points would express the total independence of the patient.
- Psychological evaluation: the Dementia Apathy Interview and Rating (DAIR) was used to assess the level of apathy of the participants. This questionnaire, which includes 14 items, was administered to a knowledgeable caregiver who had to indicate how often the patient had suffered the content of the sentence over the past month, using a four point scales: 0—no or almost never; 1—sometimes; 2—very often; 3—yes or almost always. The total score is obtained by adding the score obtained in each of the completed items and dividing it by the number of completed items. Higher scores on this scale represent greater severity of apathy. The DAIR is a reliable assessment with high internal consistency (α = 0.89) [37].
  The Yesavage scale for Geriatric Depression (EGD-15) was used to assess the presence of depressive symptoms [38,39]. This scale explores only the cognitive symptoms of a major depressive episode, with a dichotomous response pattern to facilitate the responses of the person evaluated. There is a short version of 15 items, with an internal consistency that ranges between 0.76 and 0.89. The cut-off points are: 0–5: normal; 6–10: moderate depression; >10: severe depression.

Finally, for the evaluation of anxiety and depression, the Goldberg Anxiety and Depression Scale (EADG) was used. This test consists of two subscales, one for the detection of anxiety and the other for the detection of depression. Both scales have nine questions; the first four are obligatory, while the remaining five are only formulated if any of the earlier questions are answered in the affirmative. The evaluator should ask the patient about the symptoms contained in the scales, referring to the previous 15 days. The person is considered to have anxiety if they answer affirmatively to four or more items, and depression if they give two or more affirmative answers [40].

### 2.4. Statistical Analysis

The mean and standard deviation (SD) were used to describe the sample. The change percentage of the variables between T1 and T2 in the tests was calculated as Δ (%): ((T2−T1)/T1 × 100) for each study group. Differences from T1 to T2 in each group were evaluated by the paired Student’s *t*-test or the Wilcoxon signed rank test, based on the compliance of the normality criteria of the data using the Kolmogorov–Smirnov test. Differences in Δ (%) and other tests at T1 and T2 were compared between treatment categories by the independent sample Student’s *t*-test or Mann–Whitney *U* test, with the treatment category as a fixed factor. Finally, to determine the correlation of the cognitive status and psychological status with capacity to perform basic and instrumental ADLs, the Pearson linear correlation was used, with the change percentage in these parameters.

A one-way univariate analysis of variance (ANOVA) test was used to determine significant differences in sociodemographic data at baseline. A two-way repeated measures ANOVA test was used to explore the interaction effects (time per treatment group: t×G) between both groups (control and Wii) for cognitive status, capacity to perform basic and instrumental ADLs, and anxiety, depression, and apathy. As age and sex could be factors influencing these variables, it was decided to include them as possible confounding factors in the analysis. Moreover, the statistical power was calculated.

Effect sizes were calculated using partial eta squared (η^2^ *p*) and interpreted according to the following criteria: If 0 ≤ η^2^ *p* < 0.05, there is no effect; if 0.05 ≤ η^2^ *p* < 0.26, the effect is minimal; if 0.26 ≤ η^2^ *p* < 0.64, the effect is moderate; and if η^2^ *p* ≥ 0.64, the effect is strong [41].

Statistical analysis was performed with SPSS version 25 software (IBM-Inc, Chicago, IL, USA). GraphPad Prism 6 software (GraphPad Software, Inc., San Diego, CA, USA) was used to prepare the graph. Statistical significance was established when the *p*-value < 0.05.

## 3. Results

Table 1 displays the sociodemographic data at baseline. No significant differences were determined at baseline in age, body mass, or waist, arm and leg circumferences (*p* > 0.05).

The basic and instrumental ADLs of the sample are summarized in Table 2. There were no statistically significant differences between the groups in any of the tests performed at the beginning of the study, while at the end of the study, the Wii group presented higher values than the control group on the Barthel index (66.88 ± 21.36 versus 79.25 ± 14.17) and lower values on the Katz index (1.30 ± 1.20 versus 0.82 ± 1.19). When analyzing the relationship of the anthropometric values of the same group during the study period, a statistically significant decrease in the Katz index was observed in the Wii group, as well as a statistically significant increase in the Barthel and Lawton and Brody indexes. There were statistically significant differences between groups over time on the Katz index (*p* (t×G) = 0.028), Barthel index (*p* (t×G) = 0.025), and Lawton and Brody index (*p* (t×G) < 0.001).

**Figure 1:**
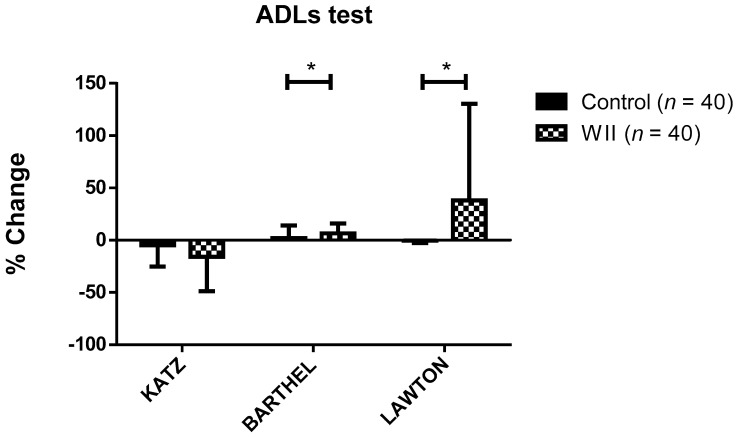
Change percentage during the study in both groups on the ADL test variables. Data are expressed as the mean ± standard deviation (SD). * Significant differences between groups (*p* < 0.001).

Change percentage on ADLs data in both groups during study is represented in Figure 1. Statistically significant differences were observed in the change percentage on the Barthel index and Lawton and Brody index during the study, depending on the group (*p* < 0.05). Specifically, in the WII group, the change percentage on the Barthel index and Lawton and Brody index was 6.61 ± 9.26% and 37.92 ± 92.51%, respectively, while in the control group it was 2.08 ± 12.03% and −0.36 ± 2.26%.

Table 3 shows the data for the cognitive status of the participants. When analyzing the cognitive status of both groups in the same study period, no statistically significant differences were observed. On the other hand, after the follow-up time, a statistically significant increase on the MCE was observed in the Wii group, and on the Fast-GDS in the control group. Furthermore, a statistically significant decrease on the MCE was observed in the control group, and on the Fast-GDS in the Wii group. There were statistically significant differences between groups over time on the MCE (*p* (t×G) = <0.001) and Fast-GDS (*p* (t×G) = 0.001).

**Figure 2:**
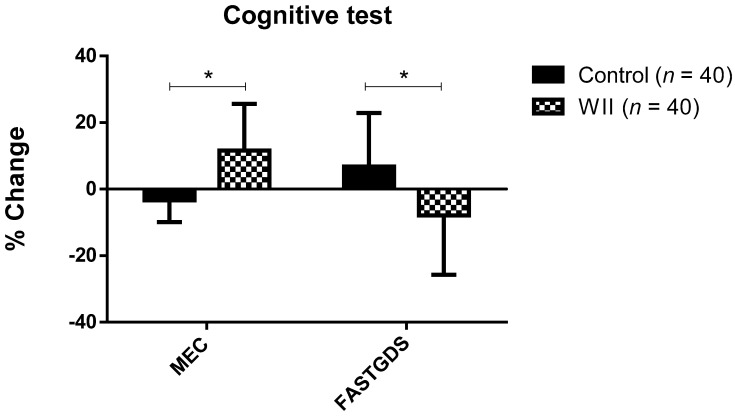
Change percentage during the study in both groups on cognitive variables; data are expressed as the mean ± standard deviation (SD); * Significant differences between groups (*p* < 0.001).

Figure 2 represents the change percentage in the cognitive variables of both groups. Statistically significant differences were observed in the change percentage on the MCE and Fast-GDS during the study, depending on the group (*p* < 0.05). Specifically, in the WII group the change percentage on the MCE and Fast-GDS was 11.64 ± 13.94%, and 7.88 ± 17.84%, respectively, while in the control group it was −3.37 ± 6.53% and 6.83 ± 16.05%.

Table 4 summarizes the psychological data of the sample. When the results obtained in the psychological tests by the same study group were compared over time, a statistically significant decrease on the EDG-15, DAIR, and EADG in the Wii group was observed, as well as a statistically significant increase on the EDG-15 and DAIR in the control group. There were no statistically significant differences between the groups in any of the tests performed at the beginning and end of the study. The joint interaction of the treatment group and time (t×G) with the results of the psychological data revealed the existence of statistically significant differences in apathy, depression, and anxiety.

**Figure 3:**
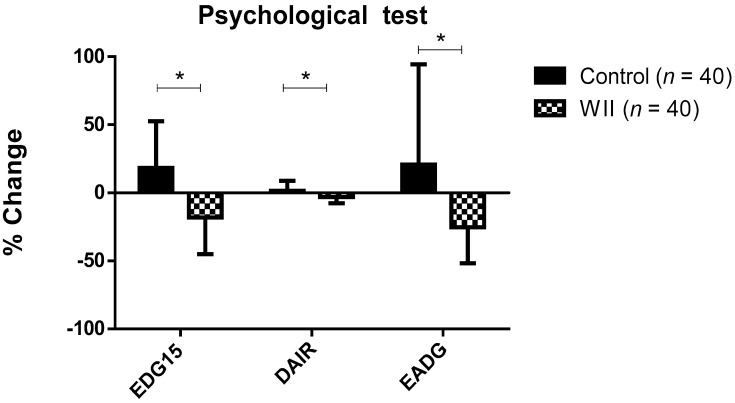
Change percentage during the study in both groups on psychological test variables; data are expressed as the mean ± standard deviation (SD); * Significant differences between groups (*p* < 0.001).

Statistically significant differences were observed in the change percentage of the psychological variables during the study, depending on the group. Specifically, when compared with the control group, the change percentage was higher and negative in the Wii group on the EDG-15 (−18.01 ± 27.11% versus 18.28 ± 34.25%), 2.00 ± 19.77%), DAIR (−3.12 ± 4.59% versus 1.43 ± 7.25%), and EADG (−24.17 ± 26.53% versus 20.75 ± 73.68%) (Figure 3).

**Figure 4:**
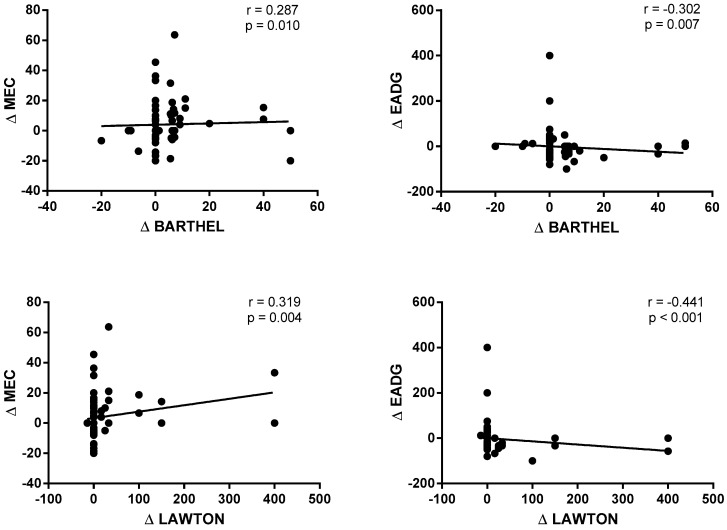
Correlations between the cognitive status and psychological status and performance on basic and instrumental activities of daily living.

The correlations between the cognitive status (MCE) and psychological status (EADG), and performance on basic and instrumental ADLs (Lawton and Brody index) are shown in Figure 4. A statistically significant and positive correlation was observed between the Δ MCE and the Δ Barthel index (r = 0.287; *p* = 0.010) and the Δ Lawton and Brody index (r = 0.319; *p* = 0.004). On the contrary, a statistically significant and negative correlation was obtained between the Δ EADG and the Δ Barthel index (r = −0.302; *p* = 0.007) and the Δ Lawton and Brody index (r = −0.441; *p* < 0.001).

## 4. Discussion

The objective of this study was determined the impact and effectiveness of use of the Wii Fit^®^ game console on improving the cognitive and psychological status of the elderly, as well as its influence and relationship with performance on basic and instrumental ADLs. The main findings of this study showed that use of Wii Fit^®^ video games improved the cognitive status of the elderly and decreased their depression levels. In addition, a relationship between the cognitive and psychological status and functionality was observed.

Age-related cognitive decline does not affect all cognitive functions equally [42]. As age advances, variability in cognitive performance is related to multiple factors [43]. The reduction in cognitive functions is associated with a poor stimulation of these capacities. The use of exergames can provide an environment that favors the activation of different sensory media [12]. Related to the results of this study, in which an improvement in the cognitive and psychological status of elderly people was observed, several authors have demonstrated an increase in executive functioning, such as information processing speed, reaction time, concentration, attention, and short-term memory, in addition to a decrease depression, anxiety, and apathy levels, in an environment of high stimulation, such as that created by exergames [44,45,46]. Santamaria et al. obtained significant improvements in attention, although not in concentration, after 15 sessions with the video game “Dance Dance Revolution” in 27 older adults (63.15 ± 5.79 years) [20]. In another study carried out with older adults with chronic schizophrenia, a significant improvement was observed in general cognitive function and on the repetition and memory subscales in a group of participants who received an intervention of 10 sessions with the video game “Bolos”, compared to the control group [47].

Regarding the psychological state, Chesler et al. observed a clear decrease in depression levels and greater social interaction after a 6-week rehabilitation program using Wii Fit^®^ games in Australian institutionalized older adults [21]. In contrast, a study carried out with 58 people who lived in a nursing home found statistically significant differences in physical appearance, but not in depression levels, after an exercise program using the Nintendo Wii Fit Plus^®^ video console [48]. These results show that the changes are probably due to the use of specific video games since, depending on which one is used, the motivational processes of the participants may increase or not. Along these lines, Jinhui Li et al. observed a positive effect of the use of the Wii Fit^®^ game console on depression, supporting its mediating role in positive emotions compared to traditional exercise [49]. In this case, exergames generated higher positive emotions than traditional exercise, further reducing depression below the threshold among older adults.

A growing number of studies are emerging that analyzed how disability influences the performance of basic and instrumental ADLs in institutionalized older people. In addition, capacity training through video games is also on the rise, since, unlike typical physical or motor therapy, video consoles not only have therapeutic benefits, but also have a motivational character which arouses interest and attention among users, favoring adherence to treatment, the achievement of objectives, and improvement of emotional well-being [50]. However, no research has specifically considered the therapeutic effect of the Wii Fit^®^ on the cognitive and psychological state of institutionalized elderly, and their influence on the performance of basic and instrumental activities of daily life. However, according to Otero et al., as in the results obtained in this study, performance on ADLs is strongly related to cognitive and psychological health in the elderly. The correct execution of ADLs is impaired by the failure of a cognitive construct domain [51]. The alteration or loss of optimal performance in ADLs can be highly disabling for the person, and it generally leads to dependency and a decreased quality of life [52].

The findings of this study must be considered within the context of its limitations. The small sample size, and selection using a non-randomized convenience sampling procedure, may lead to the results not being representative of the rest of the population. Selecting a larger sample could make the results more representative. Likewise, the small number of studies on this subject makes it difficult to compare the results we obtained. These limitations may reduce the representativeness of the findings and may have influenced the results of the study.

Even more emphasis should be placed on implementing more powerful randomized control designs with larger sample populations to test the questions of interest. There should also be greater emphasis on determining what specific musculoskeletal and neural adaptations occur in response to Wii Fit^®^ training, and how those changes compare to other commonly used balance training interventions, such as uneven balance boards and yoga.

## 5. Conclusions

In recent years, society has experienced a growing older population, so promoting quality of life in old age is an immediate challenge for social policies. Making the aging process active requires stimulation, which can be achieved through rehabilitation programs with game consoles such as the Wii Fit^®^. This device has proven to be effective in institutionalized older people, by increasing their attention and memory levels, decreasing their depression, anxiety, and apathy levels and, therefore, increasing their performance of ADLs, both basic and instrumental. Furthermore, there is a relationship between cognitive and psychological status and performance of basic and instrumental ADLs, so improving the cognitive level and reducing psychological symptoms can promote the optimal performance of ADLs.

## Figures and Tables

**Table 1:** Sociodemographic characteristics of the sample at baseline.

Control (*n* = 40)	Wii (*n* = 40)	*p*	η^2^ *p*	Statistical Power
**Age (Years)**				
83.25 ± 8.78	85.05 ± 8.63	0.285	0.015	0.186
**Body mass (kg)**				
76.35 ± 13.54	74.60 ± 13.01	0.927	0.000	0.051
**Waist circumference (cm)**				
96.73 ± 14.47	96.97 ± 14.74	0.645	0.003	0.074
**Arm circumference (cm)**				
31.18 ± 37.99	31.25 ± 37.99	0.579	0.004	0.085
**Leg circumference (cm)**				
49.86 ± 11.34	49.65 ± 12.37	0.858	0.000	0.032

Data expressed as mean ± SD; Data adjusted for sex and age. *p*: statistical significance by univariant one-way analysis of variance (ANOVA).

**Table 2:** Activities of daily living (ADLs) data of the control group and Wii group before and after the intervention.

	Control (*n* = 40)	Wii (*n* = 40)	*p*-Value (t×G)	η^2^ *p*	Statistical Power
**Katz index**					
T1	1.23 ± 1.29	0.95 ± 1.37	0.028	0.051	0.303
T2	1.30 ± 1.20	0.82 ± 1.19 * and			
**Barthel index**					
T1	68.10 ± 20.75	75.30 ± 16.69	0.025	0.064	0.614
T2	68.88 ± 21.36	79.25 ± 14.17 * and			
**Lawton and Brody index**					
T1	5.88 ± 1.81	5.25 ± 2.44	<0.001	0.161	0.964
T2	5.85 ± 1.81	5.90 ± 1.81 *			

Data expressed as mean ± standard deviation (SD); Data adjusted for sex and age; *p*-value (t×G): Group-by-time interaction (*p* < 0.05); Two-factor repeated-measures ANOVA; *: Significant differences between periods within the same study group by dependent Student’s *t*-test; significant differences between groups in the same study period by independent Student’s *t*-test.

**Table 3:** Cognitive data of the control group and Wii group before and after the intervention.

	Control (*n* = 40)	Wii (*n* = 40)	*p*-Value (t×G)	η^2^ *p*	Statistical Power
**MCE**					
T1	23.10 ± 5.73	21.28 ± 5.78	<0.001	0.369	1.000
T2	22.40 ± 6.00 *	23.32 ± 5.50 *			
**FAST-GDS**					
T1	2.82 ± 1.36	3.20 ± 1.24	<0.001	0.181	0.982
T2	3.00 ± 1.43 *	2.97 ± 1.31 *			

Data expressed as mean ± SD; Data adjusted for sex and age; *p*-value (t×G): Group-by-time interaction (*p* < 0.05); MCE: Mini-Cognitive Examination; FAST-GDS: Global Deterioration Scale; Two-factor repeated-measures ANOVA; *: Significant differences between periods within the same study group by dependent Student’s *t*-test; Significant differences between groups in the same study period by independent Student’s *t*-test.

**Table 4:** Psychological data of the control group and Wii group before and after the intervention.

	Control (*n* = 40)	Wii (*n* = 40)	*p*-Value (txG)	η^2^ *p*	Statistical Power
**EDG-15**					
T1	4.35 ± 2.80	5.85 ± 3.50	<0.001	0.335	1.000
T2	5.08 ± 3.15 *	4.55 ± 2.84 *			
**DAIR**					
T1	1.33 ± 0.26	1.42 ± 0.27	<0.001	0.180	0.980
T2	1.34 ± 0.25 *	1.37 ± 0.27 *			
**EADG**					
T1	3.47 ± 2.31	4.07 ± 2.63	<0.001	0.212	0.0994
T2	3.78 ± 2.48	3.08 ± 2.14 *			

Data expressed as mean ± SD; Data adjusted for sex and age; *p*-value (txG): Group-by-time interaction (*p* < 0.05); EDG-15: Geriatric Depression Scale; DAIR: Dementia Apathy Interview and Rating; EADG: Goldberg Anxiety and Depression Scale; Two-factor repeated-measures ANOVA; *: Significant differences between periods within the same study group by dependent Student’s *t*-test; Significant differences between groups in the same study period by independent Student’s *t*-test.

## References

[1] Bolandzadeh N., Kording K., Salowitz N., Davis J.C., Hsu L., Chan A., Sharma D., Blohm G., Liu-Ambrose T. (2015). Predicting Cognitive function from clinical measures of physical function and health status in older adults. PLoS ONE.

[2] Razani J., Casas R., Wong J.T., Lu P., Alessi C., Josephson K. (2007). Relationship between executive functioning and activities of daily living in patients with relatively mild dementia. Appl. Neuropsychol..

[3] Agüero-Torres H., Thomas V.S., Winblad B., Fratiglioni L. (2002). The impact of somatic and cognitive disorders on the functional status of the elderly. J. Clin. Epidemiol..

[4] Paixão C., Reichenheim M.E. (2005). A review of functional status evaluation instruments in the elderly. Cadernos Saude Publica.

[5] Sanchez M.A.S., Correa P.C.R., Lourenço R.A. (2011). Cross-cultural adaptation of the “functional activities questionnaire—FAQ” for use in Brazil. Dement. Neuropsychol..

[6] Gühne U., Angermeyer M.C., Riedel-Heller S. (2006). Is mortality increased in mildly cognitively impaired individuals?. Dement. Geriatr. Cogn. Disord..

[7] Black S.A., Rush R.D. (2002). Cognitive and functional decline in adults aged 75 and older. J. Am. Geriatr. Soc..

[8] Mitchell A., Savill-Smith C. (2004). The Use of Computer and Video Games for Learning: A Review of the Literature.

[9] Gee J.P. (2007). Good Video Games Plus Good Learning.

[10] Ben-Sadoun G., Sacco G., Manera V., Bourgeois J., König A., Foulon P., Fosty B., Bremond F., D’Arripe-Longueville F., Robert P. (2016). Physical and Cognitive stimulation using an exergame in subjects with normal aging, mild and moderate cognitive impairment. J. Alzheimer’s Dis..

[11] Eggenberger P., Wolf M., Schumann M., De Bruin E.D. (2016). Exergame and balance training modulate prefrontal brain activity during walking and enhance executive function in older adults. Front. Aging Neurosci..

[12] Hill N.T., Mowszowski L., Naismith S.L., Chadwick V.L., Valenzuela M., Lampit A. (2017). Computerized cognitive training in older adults with mild cognitive impairment or dementia: A systematic review and meta-analysis. Am. J. Psychiatry.

[13] Arvanitakis Z., Capuano A.W., Leurgans S.E., Bennett D.A., Schneider J.A. (2016). Relation of cerebral vessel disease to Alzheimer’s disease dementia and cognitive function in elderly people: A cross-sectional study. Lancet Neurol..

[14] Kim K., Choi B., Lim W. (2018). The efficacy of virtual reality assisted versus traditional rehabilitation intervention on individuals with functional ankle instability: A pilot randomized controlled trial. Disabil. Rehabil. Assist. Technol..

[15] Donath L., Rössler R., Faude O. (2016). Effects of virtual reality training (exergaming) compared to alternative exercise training and passive control on standing balance and functional mobility in healthy community-dwelling seniors: A meta-analytical review. Sports Med..

[16] Şimşek T.T., Çekok K. (2015). The effects of Nintendo Wii^TM^-based balance and upper extremity training on activities of daily living and quality of life in patients with sub-acute stroke: A randomized controlled study. Int. J. Neurosci..

[17] Uysal S.A., Baltaci G. (2016). Effects of Nintendo Wii™ training on occupational performance, balance, and daily living activities in children with spastic hemiplegic cerebral palsy: A single-blind and randomized trial. Games Health J..

[18] Moon J., Jung J., Cho H. (2020). Effects of balance training using a Wii Fit balance board on balance, gait and activities of daily living in patients with parkinson disease: A pilot randomized controlled trial. Med. Leg Update.

[19] Monteiro R., Da Silva L., De Tarso P., Pinheiro M., Rodrigues E., Mendes A., Lage M., Engedal K. (2017). Acute effects of exergames on cognitive function of institutionalized older persons: A single-blinded, randomized and controlled pilot study. Aging Clin. Exp. Res..

[20] Santamaría K.G., Fonseca A.S., Moncada Jiménez J., Solano Mora L.C. (2017). Balance, attention and concentration improvements following an exergame training program in elderly. Retos.

[21] Chesler J., McLaren S., Klein B., Watson S. (2015). The effects of playing Nintendo Wii on depression, sense of belonging and social support in Australian aged care residents: A protocol study of a mixed methods intervention trial. BMC Geriatr..

[22] Ritchie K., Artero S., Touchon J. (2001). Classification criteria for mild cognitive impairment. Neurology.

[23] Millán-Calenti J.C., Tubío J., Pita-Fernández S., González-Abraldes I., Lorenzo T., Maseda A. (2009). Prevalence of cognitive impairment: Effects of level of education, age, sex and associated factors. Dement. Geriatr. Cogn. Disord..

[24] Millán-Calenti J.C., Tubío J., Pita-Fernández S., González-Abraldes I., Lorenzo T., Fernández-Arruty T., Maseda A. (2010). Prevalence of functional disability in activities of daily living (ADL), instrumental activities of daily living (IADL) and associated factors, as predictors of morbidity and mortality. Arch. Gerontol. Geriatr..

[25] Franco M. (2018). Desempeño ocupacional, bienestar psicológico y sentido de la vida en personas institucionalizadas. Estudio preliminar. Rev. Psicol. Salud.

[26] Custodia N., Herrera E., Lira D., Montesinos R., Linares J., Bendezú L. (2012). Mild cognitive impairment: Where does normal ageing end and where dementia begins?. Rev. Fac. Med..

[27] Folstein M.F., Folstein S.E., McHugh P.R. (1975). “Mini-mental state”. A practical method for grading the cognitive state of patients for the clinician. J. Psychiatry Res..

[28] López J., Martí A. (2011). Instituto de Medicina Legal de Cataluña, Mini-examen cognoscitivo (MCE). Rev. Esp. Med. Leg..

[29] Reisberg B., Ferris S., Franssen E. (1985). An ordinal functional assessment tool for Alzheimer’s-type dementia. Psychiatry Serv..

[30] Beobide I., Ferro A., Miró B., Martínez S., Genua M.I. (2018). The impact of automation on the safety of drug dispensing in nursing homes. Farm. Hosp..

[31] Katz S., Ford A.B., Moskowitz R.W., Jackson B.A., Jaffe M.W. (1963). Studies of illness in the age: The index of adl, a standarized measure of biological and psychosocial function. JAMA.

[32] Silva G.D.S.F.D., Bergamaschine R., Rosa M., Melo C., Miranda R., Filho M.B. (2007). Avaliação do nível de atividade física de estudantes de graduação das áreas saúde/biológica. Rev. Bras. Med. Esporte.

[33] Mahoney F.I., Barthel D.W. (1965). Functional evaluation: The Barthel Index. Md. State Med. J..

[34] Hartigan I. (2007). A comparative review of the Katz ADL and the Barthel Index in assessing the activities of daily living of older people. Int. J. Older People Nurs..

[35] Mahoney F.I., Wood O.H., Barthel D.W. (1958). Rehabilitation of chronically Ill patients: The Influence of complications on the final Goal. South. Med. J..

[36] Lawton M.P., Brody E.M. (1969). Assessment of older people: Self-maintaining and instrumental activities of daily living. Gerontologist.

[37] Strauss E., Sperry S. (2002). An informant-based assessment of apathy in Alzheimer disease. Cogn. Behav. Neurol..

[38] Incalzi R.A., Cesari M., Pedone C., Carbonin P.U. (2003). Construct validity of the 15-Item geriatric depression scale in older medical inpatients. J. Geriatr. Psychiatry Neurol..

[39] Gómez C., Campo A. (2011). Escala de yesavage para depresión geriátrica (GDS-15 y GDS-5): Estudio de la consistencia Interna y estructura factorial. Univ. Psychol..

[40] Goldberg D., Bridges K., Duncan-Jones P., Grayson D. (1988). Detecting anxiety and depression in general medical settings. BMJ.

[41] Ferguson C.J. (2009). An effect size primer: A guide for clinicians and researchers. Prof. Psychol. Res. Pract..

[42] Harada C.N., Love M.C., Triebel K.L. (2013). Normal cognitive aging. Clin. Geriatr. Med..

[43] Lipnicki D.M., Makkar S.R., Crawford J.D., Thalamuthu A., Kochan N.A., Lima-Costa M.F., Castro-Costa E., Ferri C.P., Brayne C., Stephan B. (2019). Determinants of cognitive performance and decline in 20 diverse ethno-regional groups: A COSMIC collaboration cohort study. PLoS Med..

[44] Adcock M., Fankhauser M., Post J., Lutz K., Zizlsperger L., Luft A.R., Guimarães V., Schättin A., De Bruin E.D. (2020). Effects of an in-home multicomponent exergame training on physical functions, cognition, and brain volume of older adults: A randomized controlled trial. Front. Med..

[45] Wiloth S., Lemke N., Werner C., Hauer K. (2016). Validation of a computerized, game-based assessment strategy to measure training effects on motor-cognitive functions in people with dementia. JMIR Serious Games.

[46] Van Santen J., Dröes R.-M., Holstege M., Henkemans O.B., Van Rijn A., De Vries R., Van Straten A., Meiland F. (2018). Effects of exergaming in people with dementia: Results of a systematic literature review. J. Alzheimer’s Dis..

[47] Chan C., Ngai E., Leung P., Wong S. (2009). Effect of the adapted virtual reality cognitive training program among chinese older adults with chronic schizophrenia: A pilot study. Int. J. Geriatr. Psychiatry.

[48] Cicek A., Ozdincler A.R., Tarakci E. (2020). Interactive video game-based approaches improve mobility and mood in older adults: A nonrandomized, controlled trial. J. Bodyw. Mov. Ther..

[49] Li J., Theng Y.-L., Foo S., Xu X. (2017). Exergames vs. traditional exercise: Investigating the influencing mechanism of platform effect on subthreshold depression among older adults. Aging Ment. Health.

[50] Contreras K., Cubillos R., Hernández O., Reveco C., Santis N. (2014). Virtual rehabilitation in occupational therapy intervention. Rev. Child Radiol..

[51] Castellano J., Hurtado M.D., Contreras M.I., Pérez-Fuentes M.C., Molero Jurado M., Gázquez Linares J.J., Barragán Martín A.B., Martos Martínez A., Pérez Esteban M.D. (2016). Mantenimiento de Roles Ocupacionales en el Envejecimiento. Cuidados, Aspectos Psicológicos y Actividad Física en Relación con la Salud.

[52] Clement-Carbonell V., Ferrer-Cascales R., Ruiz-Robledillo N., Rubio-Aparicio M., Portilla-Tamarit I., Cabañero-Martínez M.J. (2019). Differences in autonomy and health-related quality of life between resilient and non-resilient individuals with mild cognitive impairment. Int. J. Environ. Res. Public Health.

